# Spatial Gradient Analysis of Single-Particle Hydration and Inter-Particle Interactions in Cement–Fly Ash–Slag System Using BSE-EDS Images

**DOI:** 10.3390/ma19102161

**Published:** 2026-05-21

**Authors:** Lixuan Mao, Zheyuan Cao, Lihui Li, Bin Zhang, Fuqiang He

**Affiliations:** 1 School of Civil Engineering and Architecture, Xiamen University of Technology, Xiamen 361024, China; lixuan.mao@xmut.edu.cn (L.M.);; 2 Intelligent Infrastructure Operation and Maintenance Technology Innovation Team of Yunnan Provincial Department of Education, Faculty of Civil Engineering and Mechanics, Kunming University of Science and Technology, Kunming 650500, China; 3 School of Engineering, Computing and Mathematics, University of Plymouth, Plymouth PL4 8AA, UK

**Keywords:** ternary cementitious systems, BSE-EDS image analysis, machine learning, calcium migration, spatial element features

## Abstract

Ion diffusion, the precipitation of hydration products, and interactions between different reactive particles are critical for optimizing the design of low-carbon cementitious systems. However, at the sub-micron scale, the complex spatial and chemical interactions among diverse components at an early age remain challenging to quantify. In this study, a machine learning-assisted BSE-EDS analytical method was applied to quantify both the phase assemblage and the spatial element features of cement–fly ash–slag ternary systems. The equidistant strip delineation of single-particle and rectangular inter-particle path methods were employed to quantify ionic diffusion gradients in the ternary systems. Single-particle strip analysis quantified the hydration front of clinker, slag and fly ash, while inter-particle analysis identified a persistent calcium-starvation zone at slag–fly ash interfaces. This region is characterized by exceptionally high Si/Ca ratios and a lower average atomic number and material density due to ionic diffusion limitations. These findings identify the slag–fly ash interface as the primary microstructural weak link, providing a robust methodology for capturing the chemical heterogeneities and optimizing the design of sustainable cementitious materials.

## 1. Introduction

The incorporation of fly ash (FA) and granulated ground blast furnace slag (GGBS) has been widely recognized as a practical and scalable pathway to achieve low-carbon cement systems [1]. The reaction kinetics of these supplementary cementitious materials (SCMs) are governed by multiple coupled processes including dissolution, ion diffusion, the precipitation of hydration products, and interactions between different reactive particles [2,3,4]. The interactions among these processes lead to the formation of a heterogeneous microstructure consisting of unreacted particles, newly formed hydration products, and evolving pore structures. Therefore, elucidating the spatial evolution of elemental distribution and phase assemblage during hydration is essential for understanding and optimizing the performance of low-carbon cementitious systems.

To quantify and understand the reaction kinetics or degree of individual components in cementitious materials, several analytical approaches have been developed. Selective dissolution methods are widely used, exploiting the differential solubility of phases in specific solvents, to allow the determination of unreacted fractions and thus the reaction extents. As standardized by RILEM TC 238-SCM, siliceous fly ash and slag can be selectively preserved using tailored chemical solutions (e.g., salicylic acid–HCl for fly ash and EDTA-TEA-DEA for slag), while dissolving clinker phases and hydration products [5,6]. Recent developments integrating ionic selective dissolution with ICP-OES analysis have further improved the accuracy and efficiency of such measurements [7]. Thermogravimetric analysis (TGA) provides another important approach, quantifying mass losses associated with hydration products such as portlandite, C-S-H, and chemically bound water, thereby enabling the evaluation of overall hydration progress [8,9,10]. Quantitative X-ray powder diffraction (XRD) Rietveld analysis combed with the partial or no known crystal structure (PONKCS) method can be used to quantify nano-crystalline phases in the SCMs-included systems [11,12]. Unfortunately, selective dissolution, XRD and TGA inherently provide bulk-averaged information and lack spatial resolution.

The image-based techniques, backscattered electron (BSE) imaging and energy-dispersive X-ray spectroscopy (EDS), have been increasingly employed to characterize phase assemblages at the microscale [13,14,15,16,17,18]. Through pixel-wise classification of elemental maps, these methods enable the estimation of phase fractions and the evaluation of the reaction degrees of individual particles [19]. Machner et al. utilized Mg/Ca versus Al/Ca relationships to characterize the rims surrounding dolomite grains, demonstrating that the Mg/Al ratio and the slope of compositional trends were sensitive to both the presence of metakaolin and the chemical environment of the exposure solution [20]. Alharbi et al. introduced confidence ellipse analysis to define statistically reliable compositional domains for phases in alkali-activated slag systems, thereby improving the robustness of phase identification [21]. Luo et al. employed ternary compositional diagrams to distinguish reaction products in alkali-activated fly ash/slag systems, successfully identifying Si-rich N-A-S-H and Al-rich N-A-S-H gel domains [22]. Despite these advances, most existing studies still rely on point-based or grid-based EDS analysis, which provides limited spatial information and is highly dependent on manual point selection.

The development of quantitative EDS (QEDS) mapping and advanced frameworks has significantly improved the reliability of phase identification in complex cementitious systems. Durdziński et al. demonstrated that integrating EDS elemental mapping enables the classification of distinct phases in fly ash and supports the quantification of their reactivity [23]. Georget et al. proposed the edxia framework to identify phases and quantify their chemical composition, particle size distributions, and volume fractions of specific phases based on SEM-EDS hypermaps [24]. It should be noted that these approaches still rely strongly on expert-defined element ratio thresholds and subjective interpretation. In recent years, machine learning has emerged as a promising tool to overcome these limitations. Machine learning-based methods, including support vector machines (SVMs) [25], K-means [17], and Gaussian mixture models (GMMs) [26], have been applied to automatically classify phases using multivariate elemental data or RGB features derived from composite EDS images. These methods improve efficiency and reduce manual intervention. Additionally, deep learning approaches, such as the QF-Net proposed by Mou et al. [27], have shown improved accuracy in microstructure segmentation tasks (e.g., microcrack detection). These developments highlight the potential of combining machine learning with BSE-EDS analysis for a more reliable and automated characterization of cementitious microstructures.

The evolution of phase assemblages during hydration is fundamentally driven by ionic transport across solid–liquid interfaces and subsequent precipitation within the pore solution [28,29]. While conventional techniques, such as TGA, XRD and selective dissolution, can track the temporal change in phase content, they rarely provide direct spatial evidence linking ion migration to the formation of hydration products. To address these challenges, the above-mentioned, machine-learning-based BSE-EDS image analysis has been recognized as a robust and efficient approach for phase segmentation in multiphase cementitious systems [15,16,17,25,26,30]. However, these studies focus on identifying phase occurrence rather than capturing the dynamic interplay between ion migration and phase assemblage. This gap is particularly critical in heterogeneous systems like cement–fly ash–slag ternary pastes. In these materials, local chemical gradients and interactions between adjacent particles are the primary determinants of microstructural development [31,32].

Based on these developments, this study proposes an integrated analytical framework that combines machine learning-assisted phase identification with quantification of both single-particle and inter-particle spatial chemical gradient. Specifically, the framework consists of three components: (1) BSE-EDS-based phase segmentation to quantify phase assemblage evolution; (2) equidistant strip analysis to resolve elemental gradients around individual clinker, fly ash, and slag particles; and (3) inter-particle path analysis to characterize chemical interactions between adjacent particles. By establishing a direct correlation between elemental distributions and phase identity, this approach aims to provide spatially resolved insights into the coupling between ion migration and phase evolution in ternary cementitious systems.

## 2. Materials, Experiments and Machine Learning-Based BSE–EDS Image Analysis

### 2.1. Materials and Sample Preparation

The binders used in this study include standard Portland cement (OPC) complying with GB8076-2008 [33], fly ash (FA), and ground granulated blast furnace slag (S). The chemical compositions of the raw materials were determined by X-ray fluorescence (XRF) using a wavelength-dispersive spectrometer equipped with a 4 kW Rh anode (PANalytical, Axios, Xiamen, China). The results are given in Table 1. The OPC is characterized by a high CaO content (63.6 wt.%), while FA is rich in SiO_2_ and Al_2_O_3_ (43.3% and 37.6%, respectively). The slag contains moderate CaO (38.6%) and notable MgO (10.1%), which is known to promote the formation of hydrotalcite-like phases during hydration. Following the Standard Test Method for Density of Hydraulic Cement (ASTM C188) [34], the measured densities for OPC, FA, and GGBS are 1.59, 1.12, and 1.51 g/cm^3^, respectively. The specific particle size distribution data for all materials are given in Table 2. The slag used in this study exhibits a D (4,3) of 17.6 μm and a span of 2.52, indicating its relatively fine nature compared to the other raw materials. The ternary system consists of 70% OPC, 15% FA, 15% GGBS (by weight). After dry-mixing for 3 min and wet-mixing for an additional 3 min, the pastes were cast and demolded after 24 h. The water-to- binder ratio was 0.4. Specimens were cured in a standard curing room (20 ± 2 °C, RH ≥ 95%) until the testing ages of 7 and 14 days.

At each age, hydration was arrested by immersing 10 mm cubic fragments in isopropanol (99.5% purity, Merck, Darmstadt, Germany) for 7 days, with the solution renewed at 12, 24, and 96 h. The samples were subsequently dried in a vacuum oven (Licheng, Xiamen, China) at 50 °C until a constant mass was achieved (variation < 0.1% per 24 h). The arrested samples were vacuum-impregnated with a low-viscosity epoxy resin (99.8%, Epofix, Struers, Ballerup, Denmark), followed by sequential grinding (SiC papers up to #2400 grit) and fine polishing with diamond suspensions (down to 0.25 µm) to achieve high-quality surfaces for SEM imaging.

### 2.2. BSE-EDS Image Acquisition and Quantification

BSE images and EDS mappings were acquired following the workflow from our previous work [15]. Firstly, the polished samples were coated with an extremely thin gold layer (~15 nm) to enhance electrical conductivity. Microstructural and chemical analyses were performed using a field-emission scanning electron microscope (CIQTEK, SEM5000Pro, Kunming, China) equipped with an Oxford Instruments EDS system (Xplore 30 mm^2^ detector, Aztec V6.1 software). The SEM was operated at an accelerating voltage of 15 kV and a working distance of 11 mm. BSE images and EDS maps were acquired at a resolution of 1024 × 704 pixels. The field of view was set at 128 × 88 μm^2^ (pixel size: 0.125 μm). During the analysis, the X-ray count rate was maintained at 80~100 kcps with a detector dead time below 20%, ensuring high signal-to-noise ratios and data accuracy. Seven key elements (Si, Al, Ca, Mg, Fe, S, and O) were mapped using the “Quantmap” module in the Aztec software to generate atomic percentage-based quantitative datasets.

**Figure 1:**
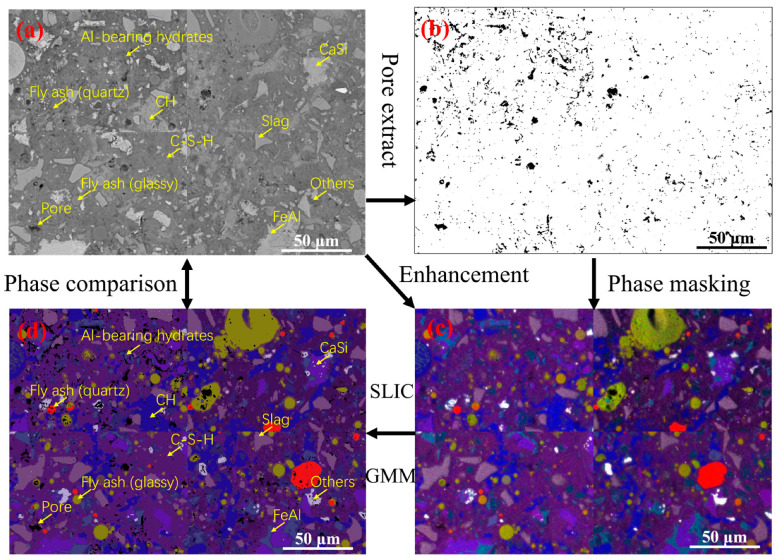
Phase identification workflow: (**a**) BSE images; (**b**) pore distribution obtained from BSE image; (**c**) enhanced composite DES images; (**d**) qualitative phase mapping.

To ensure statistical representativeness, four randomly selected fields were stitched into a 2 × 2 montage for each specimen, as shown in Figure 1. Durdziński et al. demonstrated that eight images typically provide sufficient representativeness for similar cementitious systems, achieving a maximum absolute difference in only ~3 vol.% [23]. As detailed in the Appendix A, a comparative analysis between 2 × 2 and 3 × 3 montages revealed that the 2 × 2 montage maintains the quantitative accuracy of phase area fractions within a maximum absolute difference of ~3%, confirming its suitability for capturing the heterogeneity of the ternary systems studied here.

Figure 1 presents the framework of the phase quantification. Firstly, the BSE images serve as guidance for pore segmentation via grey-value thresholding. Secondly, the BSE images under the same field of view are used as an auxiliary feature to enhance the low-resolution EDS mapping through a previously proposed guided filtering method. Then the EDS images were segmented into approximately 10,000 spatially coherent “super-pixels” using the Simple Linear Iterative Clustering (SLIC) algorithm. SLIC is a super-pixel segmentation algorithm that groups neighbouring pixels into compact, spatially coherent regions by jointly considering spatial proximity and feature similarity. In this study, it was employed to partition the enhanced EDS maps into chemically homogeneous regions, thereby reducing pixel-level noise while preserving phase boundaries.

Subsequently, a Gaussian Mixture Model (GMM), a probabilistic clustering approach, was applied to the derived features to cluster pixels into seven distinct phases: unreacted clinker (CaSi), slag, and fly ash (glassy/quartz), and hydration products including C-S-H, Calcium hydroxide (CH), Al-bearing hydrates, and Fe-rich phases (FeAl). The GMM was implemented with the following parameters: covariance type ‘full’, maximum iterations 300, convergence tolerance of 1 × 10^−3^ and initialization via k-means (10 random restarts). Feature vectors were normalized using the StandardScaler (zero mean, unit variance).

The quantitative accuracy of our BSE-EDS phase classification is supported by prior systematic validation. As detailed in our previous work [15], the results of this protocol were cross-verified with QXRD measurements and thermodynamic and microstructure simulations (VCCTL v2.0 and CemGEMS v0.8.1). These earlier comparisons demonstrated strong consistency in characterizing hydration evolution, thereby confirming the robustness of the phase quantification of the OPC–FA–slag system and the following spatial chemical analysis of individual particles and interactions between different grains.

**Figure 2:**
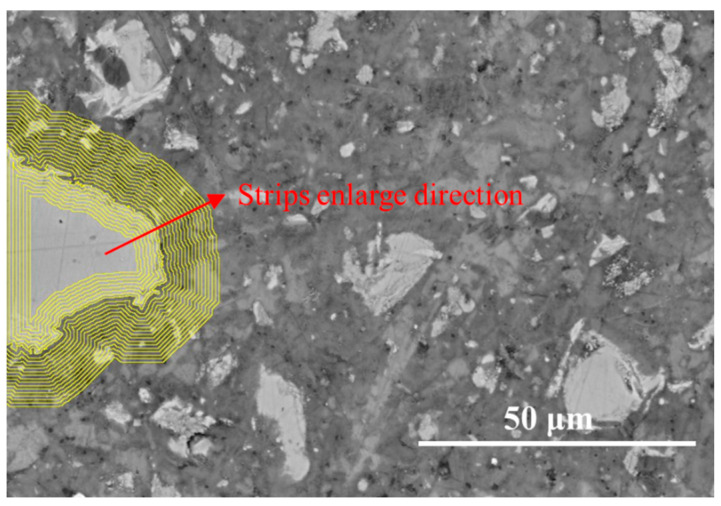
The strips delineation used for single particle analysis.

To further investigate hydration kinetics at the particle scale, spatial gradient analysis was conducted using two complementary techniques: the equidistant strip method for single-particle analysis and the rectangular transition mapping method for inter-particle interactions. Based on the elemental features within each super-pixel obtained in the quantification phase, the hydration process of individual particles was quantified using the equidistant strip method. First, the boundaries of unreacted particles (such as clinker, slag, or fly ash) were detected using the “Wand tool” in ImageJ (1.54r), which identifies contiguous regions based on intensity differences. The threshold for the Wand tool was determined empirically by adjusting the lower and upper bounds in the BSE grey-value histogram until the tool consistently traced the complete boundary of each target particle. After thresholding, the resulting particle boundaries were manually inspected on a representative subset of particles to confirm consistency with the visual identification. A detailed sensitivity analysis of threshold variation was not performed in this study, as the primary objective was to establish a reproducible workflow for spatial gradient analysis rather than to optimize boundary detection at the pixel level. Once the particle boundary was defined, successive expansion operations were applied to generate a series of concentric strips around the particle surface, as given in Figure 2. Each strip represents a narrow annular region located at a specific distance from the particle boundary. To improve data robustness, elemental intensities (Ca, Si, Al, Mg, Fe, S) and elemental ratios (e.g., Si/Ca and Al/Ca) were calculated from averaged pixel values within each equidistant strip surrounding individual particles, rather than from single-point measurements. This approach helps mitigate local fluctuations associated with EDS quantification at small scales while preserving spatially resolved trends.

**Figure 3:**
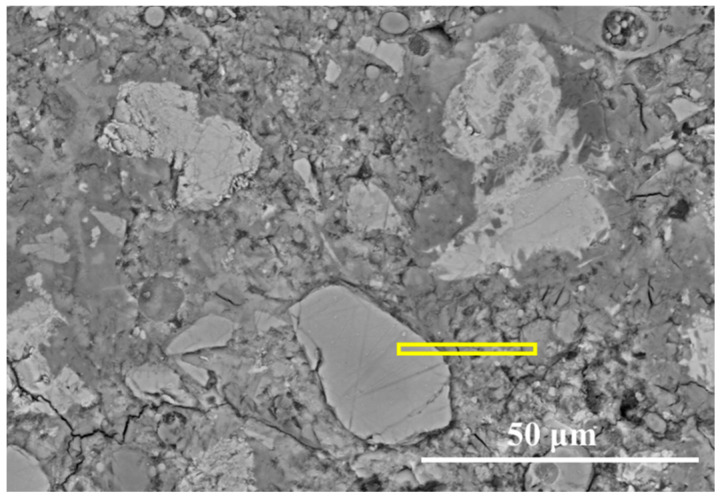
The rectangular path used for inter-particle analysis.

To quantitatively investigate the local interactions and chemical gradients between different particles, a rectangular transition mapping method was employed, as given in Figure 3. First, straight paths connecting neighbouring particle pairs, such as clinker–fly ash, clinker–slag, or slag–fly ash, were identified. Then, rectangular regions of interest (ROIs) were selected along these paths using the “Rectangle” tool in ImageJ. Approximately 4~6 candidate rectangular regions were initially identified for each system. The final representative ROI was selected based on the minimal influence from surrounding binder phases, clear geometric separation between particles, and an intermediate hydration state. Subsequently, based on the previous super-pixel segmentation results, the pixel intensities (including elemental signals, Si/Ca ratios, and BSE grey values) were extracted and averaged across the width of the rectangle at each incremental pixel along its length. This analysis allows the quantitative characterization of the interfacial transition zone (ITZ) between different particles.

## 3. Results and Discussion

### 3.1. Early Age Phase Assemblage Quantification and Element Analysis

The macroscopic performance of ternary cementitious systems is fundamentally influenced by the global evolution of their phase assemblage. The ages of 7 days and 14 days was selected as representative stages to capture the spatial evolution of elemental distributions, because, at earlier ages (e.g., 1~3 days), the secondary reactions of fly ash and slag are still limited, and the extent of ion migration and phase assemblage development may not be sufficiently pronounced for reliable spatial analysis. In addition, the relatively low mechanical strength at very early ages increases the risk of microstructural damage during epoxy resin embedding and polishing, which may compromise the accuracy of quantitative imaging. At later ages (e.g., 28 days), due to the relatively rapid hydration of clinker phases, the ion gradients and spatial heterogeneity associated with clinker dissolution may become less distinguishable, making it more difficult to resolve meaningful diffusion features.

**Figure 4:**
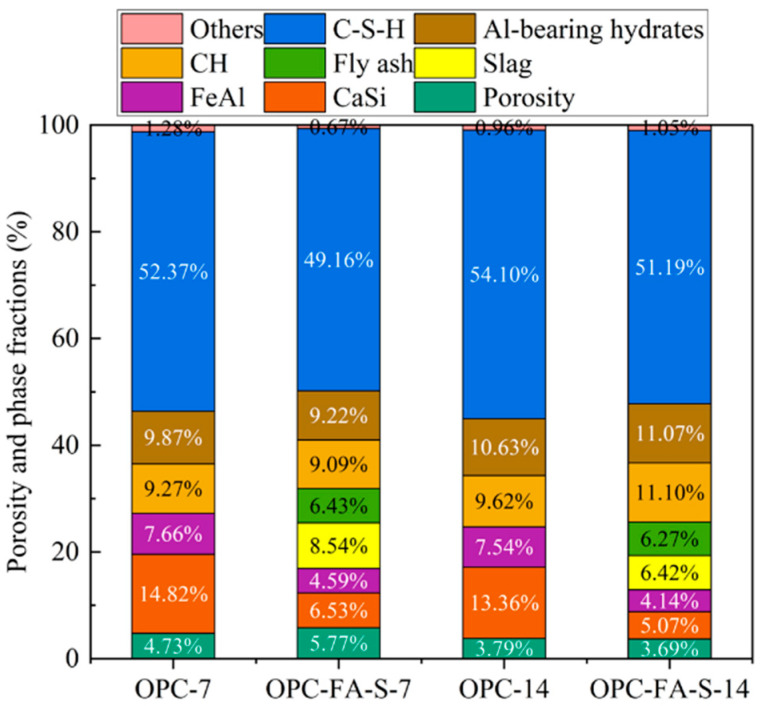
The phase quantification results of both OPC and OPC–FA–slag systems after 7- and 14-day curing.

Based on the proposed BSE-EDS quantification workflow, Figure 4 presents the transition from 7 to 14 days of hydration products in both the OPC and OPC-FA-S ternary systems. At 7 days, the C-S-H gel content in the ternary system stands at 49.16%, which is slightly lower than the 52.37% observed in the pure OPC control. This initial deficit is attributed to the “dilution effect,” where the substitution of cement with slower-reacting slag and fly ash reduces the immediate availability of clinker for hydration. However, by 14 days, the C-S-H content in the ternary system increases to 51.19%, narrowing the gap significantly. This growth is accompanied by a substantial increase in Al-bearing hydrates (rising from 9.22% to 11.07%), which is more than double the amount found in the OPC control (4.86%). The synergistic dissolution of slag and fly ash provides a surplus of reactive alumina, leading to the formation of Al-bearing phases that reinforce the matrix. Moreover, at 14 days, the porosity of the OPC-FA-S system drops dramatically to 3.69%, effectively surpassing the density of the pure OPC system (3.79%). This quantitative evidence proves that while SCMs delay early hydration, their mid-to-long-term pozzolanic and hydraulic contributions result in a much more refined pore structure.

**Figure 5:**
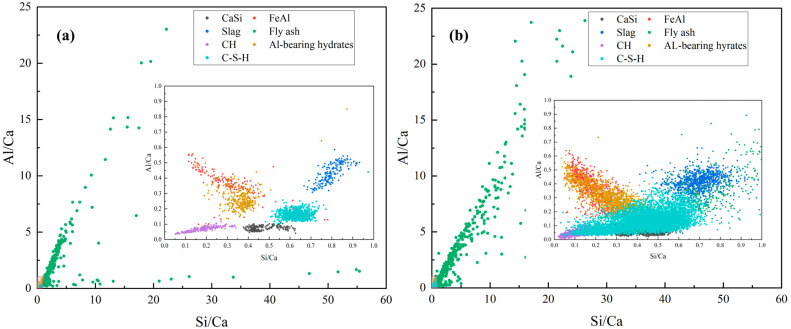
The elemental features of main phases in OPC-FA-Slag system after (**a**) 7-day curing, (**b**) 14-day curing.

The microchemical evolution of the OPC-FA-S ternary system is captured in the Si/Ca versus Al/Ca scatter plots derived from the quantitative EDS analysis, as shown in Figure 5. The central cluster in both plots represents the C-S-H gel, the primary binder responsible for the structural integrity of the paste. At 7 days, the cluster is densely populated within the range of Si/Ca ≈ 0.6~0.7 and Al/Ca ≈ 0.1~0.15. Compared to the theoretical Si/Ca ratio of 0.45~0.50 for OPC systems, the observed higher silica content indicates that silica released from slag and fly ash has already been incorporated into the C-S-H structure [35,36]. At 14 days, the cluster shifts slightly toward both higher Si/Ca and higher Al/Ca values, indicating further structural modification of the C-S-H phase. These observations are consistent with the mechanism of secondary pozzolanic reactions, where Si and Al ions released from SCMs react with the calcium hydroxide (CH) generated from cement hydration, leading to the precipitation of additional calcium C-S-H gels [37,38]. As hydration progresses, Al ions are incorporated into the silicate structure through the isomorphous substitution of Al for Si, a process that preferentially occurs at the bridging tetrahedral sites of the dreierketten silicate chains [39].

The region near the origin, Si/Ca ≈ 0 and Al/Ca ≈ 0, corresponds to the calcium hydroxide (CH) phase. A comparison between the 7-day and 14-day plots reveals a noticeable reduction in the density of points in this region. This trend indicates the progressive consumption of CH through pozzolanic reactions involving both slag and fly ash. The scatter points located directly above the C-S-H cluster (Al/Ca > 0.3) correspond to Al-bearing hydrates, such as ettringite (AFt) or monosulfoaluminate (AFm). At 14 days, this region becomes more populated and distinct. The release of aluminum from the slag glass drives the formation of these secondary crystalline phases. Their presence is instrumental in refining the pore structure by filling capillary voids.

The green points located in the high-ratio region (Si/Ca > 2.0) represent the silica–alumina-rich glassy phases of fly ash. The reduction in the density of these high-coordinate points at 14 days reflects the gradual dissolution of the FA surface. In contrast, slag particles tend to appear in a moderately elevated compositional region, typically around Si/Ca ≈ 0.8~1.2 and Al/Ca ≈ 0.3, reflecting their higher calcium content relative to fly ash. From 7 to 14 days, the contraction and dispersion of this cluster suggest the relatively high early reactivity of slag, which actively dissolves and contributes Ca, Si, and Al species to the hydration system.

### 3.2. Single-Particle Spatial Hydration Quantification

**Figure 6:**
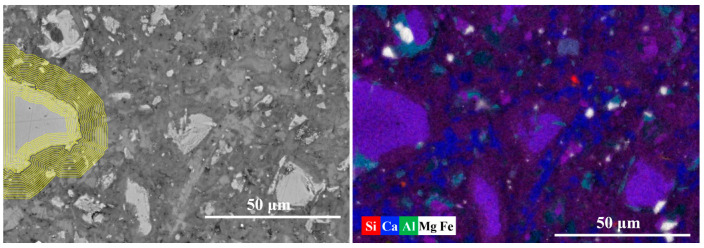
The equidistant strips (yellow strips in BSE image) applied for spatial elemental analysis of clinker particle.

**Figure 7:**
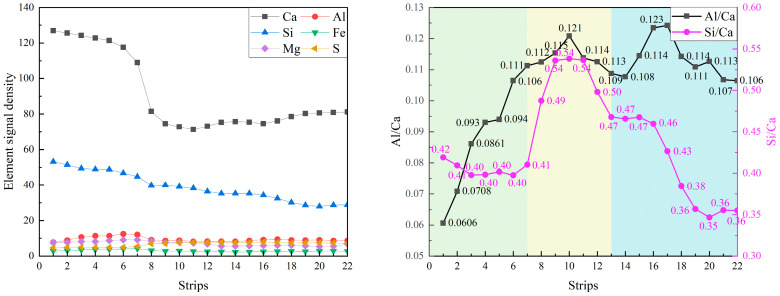
The element signal density and element ratios against the distance from the clinker particle.

To understand the hydration kinetics at the sub-micron scale, the equidistant-strip method was applied to quantify the chemical evolution from the core of the particles outward to the bulk matrix. Figure 6 presents the equidistant strips applied to the spatial-elemental analysis of clinkers after 7 days of curing. Figure 7 plots the elemental signal density profiles and the corresponding atomic ratio curves of the region surrounding the clinker particle.

The anhydrous core (strips 1~7) is characterized by a high-intensity plateau of Ca and Si, representing the unreacted C_2_S/C_3_S. A critical transition occurs at strip 8, where the Ca signal density presents a sharp drop, which represents the physical boundary of the original clinker grain and the inner hydration product (IP) zone. Between strips 8 and 12, pronounced drop in Ca intensity occurs, accompanied by a moderate decrease in Si. This transition zone likely represents the inner hydration product region formed adjacent to the clinker surface. Here, the Al/Ca ratio increases to its maximum (0.121) and the Si/Ca ratio also peaks (0.54). According to LHôpital’s research, the Al/Si ratio in C-S-H is limited to approximately 0.15 under equilibrium conditions. Converting our measured Al/Ca ratio of 0.121 to Al/Si using the local Si/Ca ratio of 0.54 (Al/Si = (Al/Ca)/(Si/Ca)) yields Al/Si ≈ 0.22, which slightly exceeds this upper limit. This suggests that a portion of the aluminum detected in this region may reside in secondary Al-bearing phases (e.g., AFm/AFt) [40]. This can also be observed in the composite EDS mapping; the cyan part around the clinker core represents the Al-bearing phase as given in Figure 6. Notably, the Al intensity in this zone is significantly higher than that found in pure OPC systems, suggesting that aluminum ions, dissolved from slag and fly ash, have successfully migrated through the pore solution to be incorporated into the hydration products during early hydration.

Further away from the clinker surface (strips 13~22), the Ca signal becomes relatively stable, whereas the Si/Ca ratio gradually decreases from approximately 0.50 to about 0.35. The reduction in the Si/Ca ratio may indicate the coexistence of calcium-rich phases such as CH. Meanwhile, the Al/Ca ratio remains relatively high (around 0.11) and even exhibits a local maximum near Strip 17. This radial analysis quantitatively proves that the chemical modification in ternary systems is a global phenomenon, not limited to the SCMs–matrix interface.

**Figure 8:**
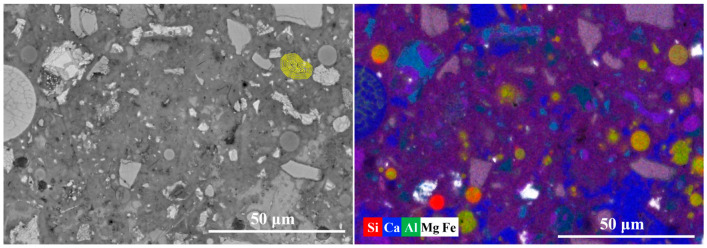
The equidistant strips (yellow strips in BSE image) applied for spatial elemental analysis of fly ash particle.

**Figure 9:**
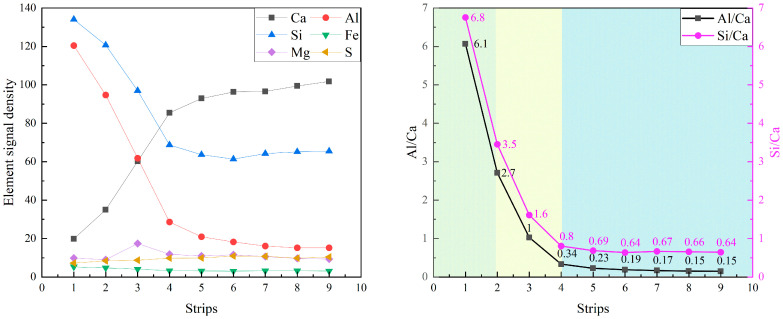
The element signal density and element ratios against the distance from the fly ash particle.

Figure 8 presents the equidistant strips applied to the spatial-elemental analysis of a FA grain after 7 days of curing. A small particle has been chosen to reflect the early-age reactivity of FA. Based on the EDS strip analysis, the region surrounding the fly ash particle can be divided into three zones: the fly ash core, the reaction front, and the surrounding matrix, as given in Figure 9.

In the core region of the fly ash particle (Strips 1~2), the elemental signals are dominated by Si and Al, with Si/Ca reaching approximately 6.8 and Al/Ca around 6.1. The high ratios confirm that the fly ash core remains largely unreacted after 7 days of hydration. The reaction front (strips 2~4) shows an aggressive “double diffusion” process: Ca^2+^ from the pore solution migrates inward while Si and Al species released from the FA glass migrate outward. This process represents the typical pozzolanic reaction mechanism, in which fly ash consumes calcium hydroxide and produces Al-included C-S-H gel. Beyond the reaction front, in the matrix region (Strips 4~9), the elemental distribution becomes relatively stable. Meanwhile, the Al/Ca ratio ranges from 0.15 to 0.23, significantly higher than that around clinker particles as observed in Figure 7. These observations demonstrate that, even at 7 days of hydration, fly ash does not merely act as an inert filler but actively participates in chemical reactions.

**Figure 10:**
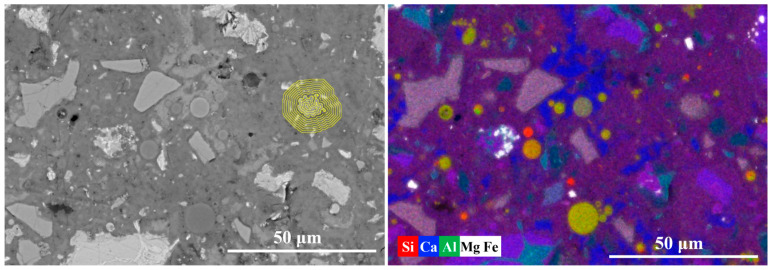
The equidistant strips (yellow strips in BSE image) applied for spatial elemental analysis of slag particle.

**Figure 11:**
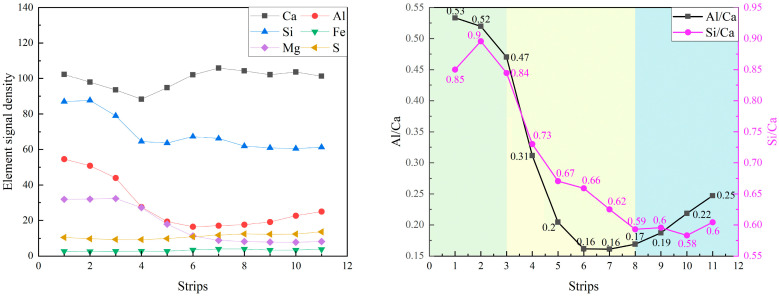
The element signal density and element ratios against the distance from the slag particle.

Figure 10 presents the equidistant strips applied to the spatial-elemental analysis of slag particle after 7 days of curing. Slag is a latent hydraulic material containing significant amounts of Ca, Si, Al, and Mg, which allows it to participate more actively in early hydration reactions. As shown in Figure 11, within the slag particle core (strips 1~3), the elemental signals for Ca, Si, Al, and Mg are all relatively high and stable. In particular, the Mg signal is significantly stronger than in the clinker or fly ash systems. The Si/Ca ratio in this region ranges from approximately 0.85 to 0.90, while the Al/Ca ratio is around 0.53. These values correspond well with the typical chemical composition of ground granulated blast furnace slag.

In the reaction layer (strips 4~8), all major elemental signals decrease simultaneously, indicating the dissolution of the slag glass structure. Meanwhile, both the Al/Ca ratio and Si/Ca ratio decrease sharply in this region, which indicates that calcium ions supplied by the surrounding cement matrix participate in the formation of hydration products. In the outer matrix region (Strips 8~11), the Al/Ca ratio exhibits a slight increase from 0.17 to around 0.25. The increase in Al/Ca suggests the formation of aluminum-rich phases in the vicinity of slag particles, which is consistent with the phase identification in the composite EDS mapping in Figure 10. These phases contribute to a more tortuous pore structure and higher early-age strength. Overall, the results indicate that slag exhibits higher early reactivity compared with fly ash.

### 3.3. Inter-Particle Interactions Quantification

**Figure 12:**
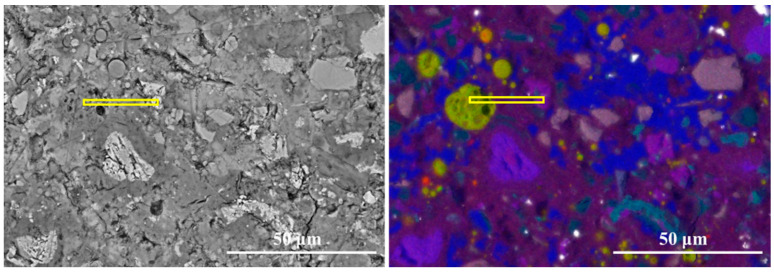
The rectangular path (yellow rectangle) applied for spatial element analysis between fly ash and clinker particles.

**Figure 13:**
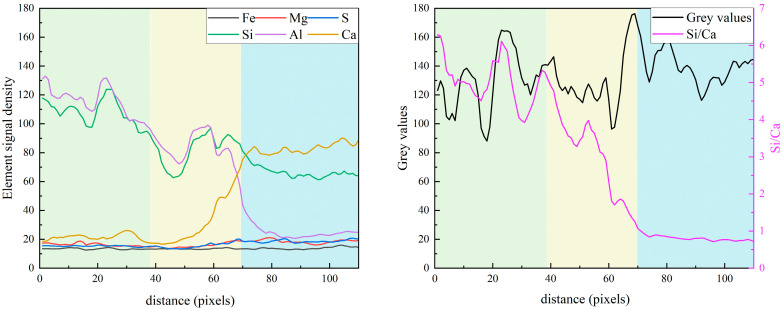
The element signal density, element ratios, and grey values between fly ash and clinker particles.

Figure 12 presents the rectangular path applied to the inter-particle analysis between FA and clinker particles after 7 days of curing. Along the path from the fly ash core to the clinker side, significant chemical gradients are observed on Figure 13. Within the fly ash region, the signals of Si and Al are very high, while Ca remains extremely low, resulting in a Si/Ca ratio exceeding 6.0. This confirms the aluminosilicate nature of the fly ash particle [41]. In the interfacial transition zone (ITZ), Si and Al intensities decrease sharply, whereas the Ca signal increases dramatically due to the diffusion of calcium ions from the hydrated cement matrix. Moreover, a distinct “valley” in grey values occurs between the clinker and FA particles, coinciding with a rise in the Si/Ca ratio. This suggests that the ITZ between clinker and fly ash is a region of lower average atomic number and local material density. This trend is consistent with observations by He et al. in CO_2_-cured cement–fly ash–slag systems, where the ITZ has not yet produced sufficient hydration products to effectively fill and densify the inter-particle space [32]. Further toward the clinker side, the Ca signal stabilizes at a high level while the Si/Ca ratio remains between 0.7 and 0.8. The grey value also increases again, indicating a mixing of unreacted clinkers and C-S-H gel.

**Figure 14:**
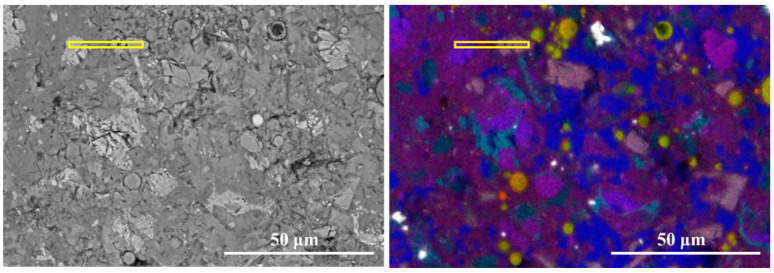
The rectangular path (yellow rectangle) applied for spatial element analysis between clinker and slag particles.

**Figure 15:**
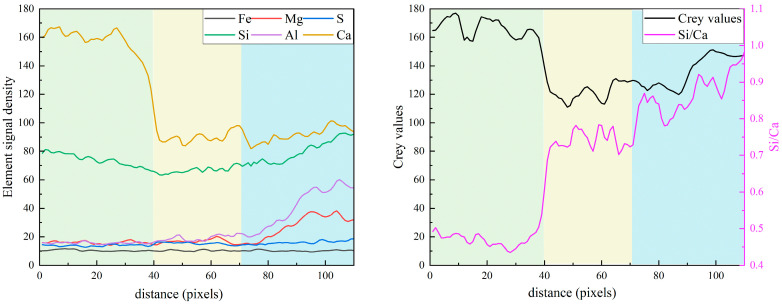
The element signal density, element ratios, and grey values between clinker and slag particles.

Figure 14 presents the rectangular path applied to the inter-particle analysis between clinker and slag particles after 7 days of curing. Compared with the clinker–fly ash interface, the clinker–slag interface shows a more gradual chemical transition in Figure 15. In the clinker core region, Ca signals are extremely high, and the Si/Ca ratio remains around 0.4~0.5. As the path moves into the hydration product region, the Ca signal decreases while the Si/Ca ratio increases to approximately 0.7~0.8, indicating the formation of the C-S-H gel. Approaching the slag-influenced region, the signals of Mg and Al increase significantly, demonstrating the participation of slag dissolution products. The results show that the clinker–slag interface exhibits higher Si/Ca ratio and grey values than the clinker–fly ash interface, indicating a more chemically evolved and locally densified interfacial region. This behaviour is closely related to the higher early-age reactivity of slag compared with fly ash. Similar observations were reported by Zhang et al., who found that cement–GGBS paste exhibited a higher maximum hydration temperature (39.8 °C) than cement–FA paste (37.0 °C), reflecting more intense and sustained early hydration reactions in slag-containing systems [42].

**Figure 16:**
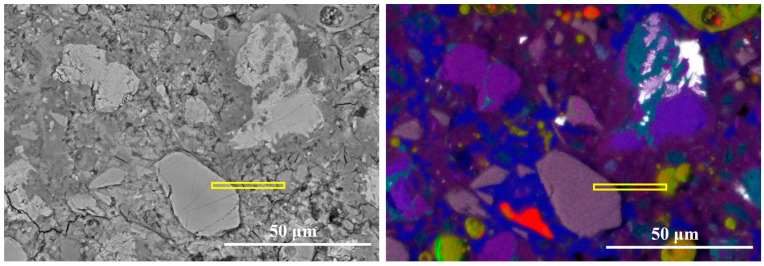
The rectangular path (yellow rectangle) applied for spatial- element analysis between slag and fly ash particles.

**Figure 17:**
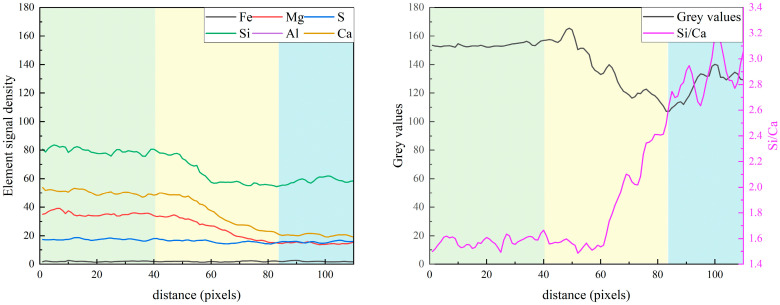
The element signal density, element ratios, and grey values between slag and fly ash particles.

Figure 16 presents the rectangular path applied to the inter-particle analysis between slag and FA particles after 7 days of curing. As shown in Figure 17, the elemental distribution along the path connecting slag and fly ash particles reveals a distinct chemical environment characterized by limited calcium availability. In the core regions of both the fly ash and slag particles, the intensities of all major elemental signals remain virtually constant. This stability indicates a low degree of dissolution for both SCMs during the early hydration stage, a phenomenon that is particularly pronounced when the local micro-environment fails to provide sufficient calcium ions to trigger the pozzolanic or latent hydraulic reactions. Moving toward the interfacial transition zone (ITZ), a sharp decrease in the Ca and Mg signals is observed, accompanied by a gradual reduction in grey values. This suggests a competitive consumption of calcium ions at the slag–FA interface and the formation of less dense, compositionally evolving hydrates. Existing research has also showed that calcium availability is critical for governing the reaction kinetics and gel formation in SCMs-included systems [22]. Limited Ca^2+^ diffusion leads to the coexistence of two chemically distinct gels, and incompatibility between these gels results in microcracks along their boundaries. This observation is analogous to the calcium starvation zone at the slag–FA interface in our ternary system, where both SCMs consume Ca^2+^, leading to a weak interfacial zone. Addressing the Ca^2+^ diffusion limit at these interfaces offers a critical pathway for improving the mechanical and chemical continuity of the ternary cementitious systems.

## 4. Conclusions

This study quantified the early-age microstructural evolution of an OPC-FA-S ternary system using a combination of machine learning-assisted BSE-EDS quantification method and spatial gradient analysis. Based on the phase identification and the sub-micron chemical gradients of clinker, FA, and slag, the following conclusions can be drawn:

1. The equidistant strip analysis reveals clear spatial gradients from unreacted particle cores to the surrounding hydration products. Around the clinker particles, a layered structure is observed, consisting of a Ca-rich core, an Al- and Si-enriched inner product region, and a relatively Ca-dominated outer product. These gradients reflect coupled processes of dissolution, ion diffusion, and precipitation during early hydration.
2. At 7 days, the FA particles exhibit high chemical stability and remain largely unreacted, with the anhydrous core maintaining an extremely high Si/Ca ratio (>6.8). Slag demonstrates a significantly higher early-stage reactivity than FA. The radial elemental profiles show a gradual dissolution of the slag glass, accompanied by a synchronized enrichment of Mg and Al at the interface.
3. The continuous variation in elemental signals and the Si/Ca and Al/Ca ratios across the interface indicates ion exchange between the clinker and the SCMs. Clinker grains serve as the primary Ca^2+^ hubs, establishing a concentration gradient that drives the pozzolanic reactions of the SCMs.
4. The interaction between the two SCMs represents the most critical microstructural link. As both slag and FA are calcium consumers, the inter-particle zone suffers from localized calcium deficiency. This region is characterized by an exceptionally high Si/Ca ratio (>2.4) and the lowest grey value across the entire matrix.
5. The integrated approach of machine learning-assisted BSE-EDS imaging analysis, equidistant strip, and path analysis provides a high-resolution tool for quantifying the ITZ and hydration fronts. The calcium migration limit at the SCM particle interface can be served as a critical entry point for further enhancing the performance of ternary systems.
6. The proposed framework will be further extended to less-characterized or emerging cementitious systems. In such cases, an adaptive determination of the cluster number, combined with compositional feature analysis, will be explored to enhance phase identification and potentially enable the discovery of previously unrecognized hydration products.

## Figures and Tables

**Table 1:** Chemical composition of OPC, FA, and slag, as determined by XRF [wt.%].

	CaO	SiO_2_	Al_2_O_3_	Fe_2_O_3_	MgO	Na_2_O	K_2_O	SO_3_	TiO_2_	Others
OPC	63.6	14.2	5.2	4.2	3.9	1.7	1.4	3.1	0.7	1.4
FA	4.4	43.3	37.6	6.3	0.9	2.1	1.1	1.3	1.4	1.6
Slag	38.6	29.1	14.6	0.4	10.1	1.6	0.6	2.0	1.3	1.7

**Table 2:** Particle size distribution of OPC, FA, and Slag, as determined by laser diffraction (µm).

	D_10_	D_50_	D_90_	Volume Mean Diameter D (4,3)	Span
OPC	3.2	18.0	52.5	23.7	2.74
FA	4.6	21.1	82.0	35.1	3.67
Slag	2.8	12.8	35.0	17.6	2.52

**Figure A1:**
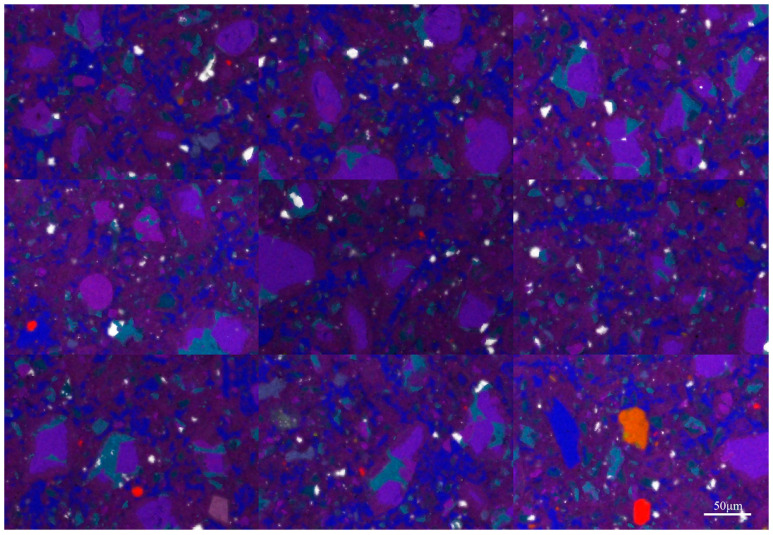
The illustration of image stitching of 14-day curing OPC system.

**Figure A2:**
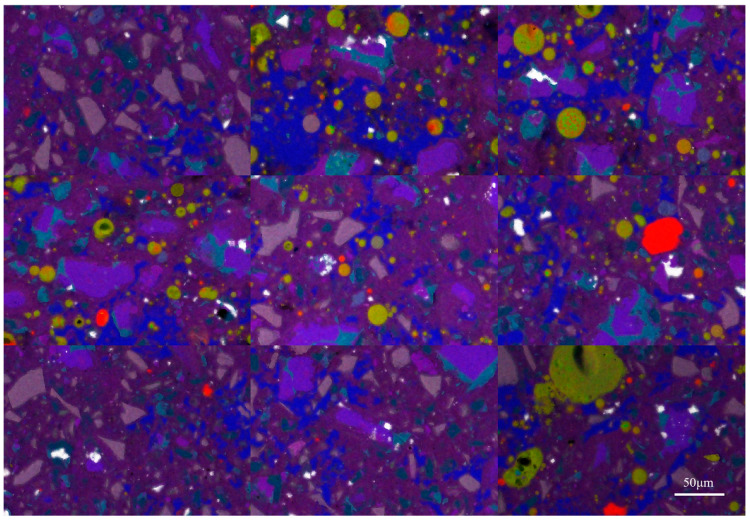
The illustration of image stitching of 14-day curing OPC–FA–slag system.

**Figure A3:**
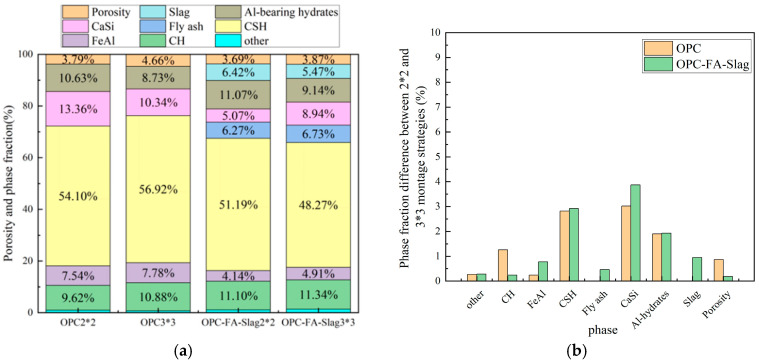
Comparison of (**a**) phase compositions and (**b**) absolute difference in phase fractions between the 2 × 2 and 3 × 3 montage strategies.

## Data Availability

The original contributions presented in this study are included in the article. Further inquiries can be directed to the corresponding author.

## Appendix A. Verification of Statistical Representativeness of the 2 × 2 Montage

To address concerns regarding dataset size and statistical representativeness, a comparative analysis was conducted to evaluate the impact of the field of view on quantitative phase analysis results. Durdziński et al. demonstrated that eight images typically provide sufficient representativeness for similar cementitious systems, achieving a maximum absolute difference in only ~3 vol.% [23]. Therefore, a comparison between the standard 2 × 2 montage (four random regions) and a larger 3 × 3 montage (nine random regions) was conducted.

The verification was performed on Ordinary Portland Cement (OPC) and ternary cement (OPC–FA–slag) systems at 14 days of age, as shown in Figure A1 and Figure A2. Figure A3a presents the area fractions of phase compositions for different systems using both strategies. It is observed that the contents of various phases (e.g., C-S-H, unhydrated cement and porosity) derived from 2 × 2 and 3 × 3 montages are highly consistent. Figure A3b quantifies the absolute differences between the two strategies. The results show that the deviations in volume fractions for all major phases are minimal (maximum absolute difference < 3 area.%). Meanwhile, while a larger montage (3 × 3) offers broader spatial coverage, it significantly increases computational costs and data processing complexity. Each sampling region in this study was acquired at an extremely high resolution (approx. 1024 × 704 pixels, pixel size ≈ 0.125 μm). This means a single sample for 3 × 3 montage contains over 6 million pixels for quantification analysis. The 2 × 2 strategy achieves an optimal balance between precision and efficiency by ensuring microstructural coverage while avoiding massive data storage and computational burden.
